# Delayed Recovery of a Disintegrin-Like and Metalloproteinase With Thrombospondin Type 1 Motif 13 (ADAMTS13) Activity in Immune Thrombotic Thrombocytopenic Purpura Treated With Caplacizumab and Rituximab

**DOI:** 10.7759/cureus.78884

**Published:** 2025-02-12

**Authors:** Yusuke Fujiwara, Muneo Okamoto

**Affiliations:** 1 Department of Hematology, Shonantobu General Hospital, Chigasaki, JPN; 2 Department of Hematology, Nippon Medical School, Tokyo, JPN

**Keywords:** adamts13, adamts13 inhibitor levels, caplacizumab, plasma exchange therapy, plasmic score, rituximab (rtx), thrombotic thrombocytopenic purpura (ttp)

## Abstract

An 81-year-old man was admitted with fever, impaired consciousness, thrombocytopenia, and hemolytic anemia after severe acute respiratory syndrome coronavirus 2 infections. Laboratory findings showed reduced a disintegrin-like and metalloproteinase with thrombospondin type 1 motif 13 (ADAMTS13) activity with the presence of ADAMTS13 inhibitor, confirming immune thrombotic thrombocytopenic purpura (TTP). Initial treatment with plasma exchange (PE), corticosteroids, and caplacizumab attenuated symptoms; however, ADAMTS13 activity remained low. Refractory TTP was managed with rituximab. Thrombocytopenia recurred during treatment, indicating relapse, which was treated with additional PE, corticosteroids, caplacizumab, and rituximab. The patient achieved remission on day 57. The careful monitoring of rituximab efficacy is necessary because caplacizumab may delay ADAMTS13 recovery.

## Introduction

Immune thrombotic thrombocytopenic purpura (iTTP) is a type of thrombotic microangiopathy that is caused by the formation of autoantibodies against a disintegrin-like and metalloproteinase with thrombospondin type 1 motif 13 (ADAMTS13) [1]. The incidence of thrombotic thrombocytopenic purpura (TTP) has been reported to be 1.74 per million population. The median age at onset in Japan is 54 years, and women account for 55% of cases. TTP is a life-threatening disease with a mortality rate of >90% without proper treatment, necessitating a prompt diagnosis and appropriate management [2]. Treatment consists of immediate plasma exchange (PE) [3,4], and corticosteroids can be omitted to suppress ADAMTS13 inhibitors [5]. Caplacizumab is a recombinant single-chain bivalent humanized monoclonal antibody that targets the A1 domain of von Willebrand factor (VWF). It inhibits ultrahigh-molecular-weight VWF-mediated platelet aggregation, which is a hallmark of TTP, by blocking the interaction between VWF and platelets. The addition of caplacizumab has been shown to facilitate early symptom improvement [6-8].

However, some TTP cases relapse or become refractory and are treated with rituximab, an anti-CD20 monoclonal antibody. Rituximab typically exerts therapeutic effects within approximately two weeks [9,10]. The recovery of ADAMTS13 activity was previously shown to be delayed in TTP patients treated with caplacizumab [11,12]. This case report describes a patient with relapsed and refractory TTP who was treated with rituximab and caplacizumab, achieving remission on day 57. However, ADAMTS13 activity recovery was delayed. In relapsed and refractory TTP treated with rituximab and caplacizumab, the recovery of ADAMTS13 activity may be delayed, necessitating caution when evaluating treatment outcomes.

## Case presentation

An 81-year-old man was brought to our hospital with impaired consciousness. He was previously diagnosed with ocular myasthenia gravis in August 2023, which improved with ambenonium 10 mg/day. In mid-February 2024, the patient was admitted to a local hospital, presenting with fever and fatigue. Severe acute respiratory syndrome coronavirus 2 (SARS-CoV-2) was detected in a nasopharyngeal swab via immunochromatography. Acetaminophen was prescribed, and fever and fatigue resolved within a few days. However, consciousness became impaired three weeks later, and the patient visited our hospital in March 2024.

**Figure 1:**
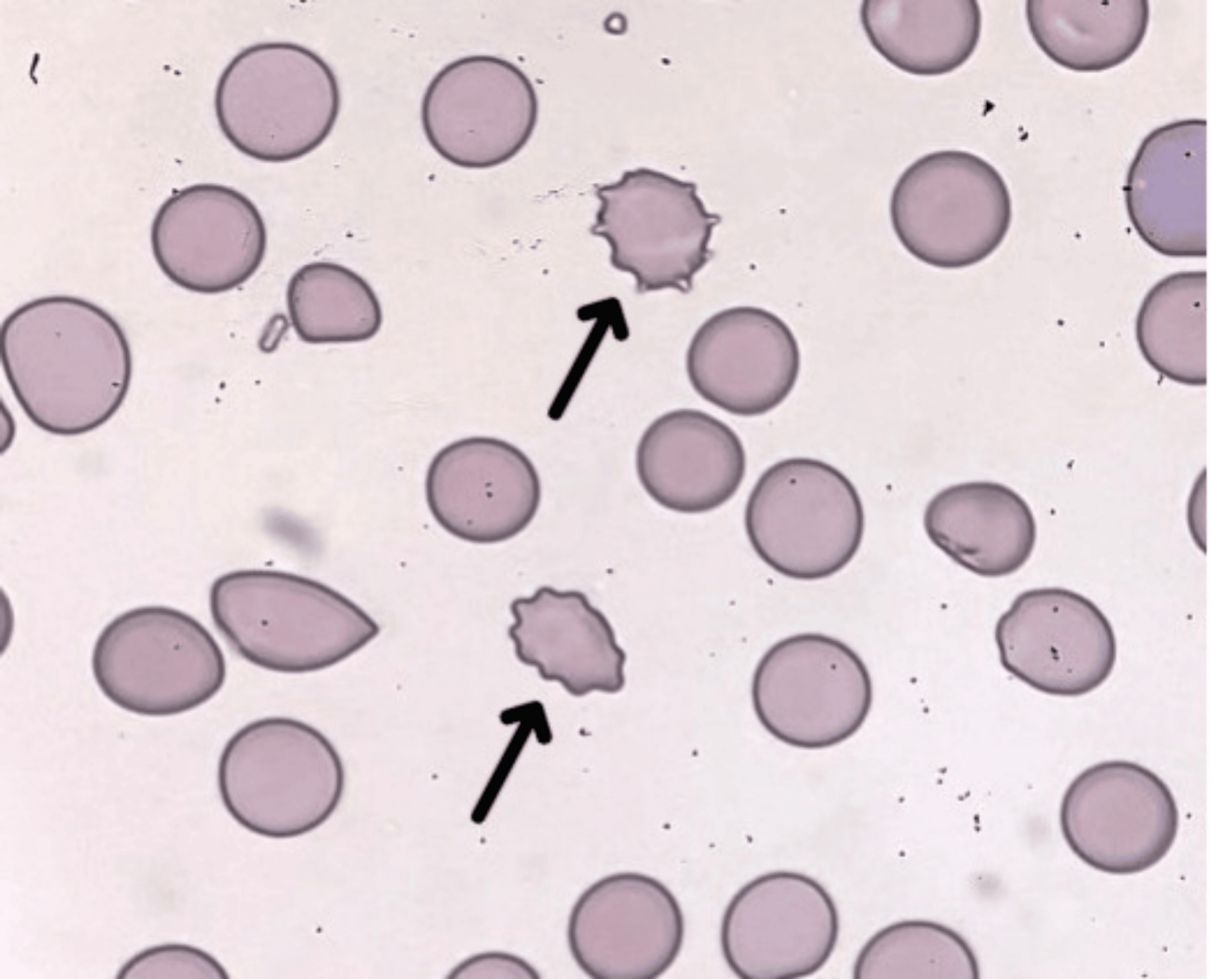
Peripheral blood smear of the patient on admission, demonstrating schistocytes (arrows), which accounted for 0.8% of the total red blood cells

The patient presented with impaired consciousness (Glasgow Coma Scale: E3 V2 M5), fever of 40.2°C, generalized jaundice, and multiple subcutaneous hemorrhages. Laboratory findings on admission are shown in Table 1, and peripheral blood smears on admission are shown in Figure 1.

**Table 1:** Laboratory data on admission SARS-CoV-2: severe acute respiratory syndrome coronavirus 2; APTT: activated partial thromboplastin time; CLβ2GPI: cardiolipin-beta2 glycoprotein 1 complex antibody; BU: Bethesda unit; IU: international units

Parameter	Patient value	Reference range	Unit
Complete blood count			
White blood cell	8,160	3,300-8,600	/μL
Red blood cells	227	435-555	×10⁴/μL
Reticulocyte	0.8	0.2-2.7	%
Schistocyte	0.8	0	%
Hemoglobin	7.5	13.7-16.8	g/dL
Platelet	1.4	15.8-34.8	×10⁴/μL
Biochemical tests			
Total protein	6.5	6.6-8.1	g/dL
Albumin	3.3	4.1-5.1	g/dL
Aspartate aminotransferase	45	13-30	U/L
Alanine aminotransferase	14	10-42	U/L
Total bilirubin	6.3	0.4-1.5	mg/dL
Direct bilirubin	0.8	<0.4	mg/dL
Indirect bilirubin	5.5	<0.8	mg/dL
Alkaline phosphatase	62	38-113	U/L
Creatine phosphokinase	194	59-248	U/L
Lactate dehydrogenase	1,016	124-222	U/L
Blood urea nitrogen	42.9	8.0-20.0	mg/dL
Creatinine	1.05	0.65-1.07	mg/dL
Sodium	148	138-145	mmol/L
Potassium	3.8	3.6-4.8	mmol/L
Chloride	107	101-108	mmol/L
C-reactive protein	3.65	0.00-0.14	mg/dL
Haptoglobin	1.0	19-170	mg/dL
Direct Coombs test	(-)	(-)	-
Cold agglutinin titer	16	<64	-
Vitamin B12	811	180-914	pg/mL
Folic acid	5.0	3.1-19.9	ng/mL
Antinuclear antibody	80	<40	-
Rheumatoid factor	26.9	0-15	U/mL
SARS-CoV-2 antigen	(-)	(-)	-
Blood coagulation test			
Prothrombin time	13.9	8.0-12.0	seconds
APTT	31.7	26.0-38.0	seconds
Fibrinogen	306	170-410	mg/dL
Fibrin degradation products	7.7	<5.0	μg/mL
ADAMTS13 activity	8	>10	%
ADAMTS13 inhibitor	1.9	<0.5	BU/mL
Lupus anticoagulant	1.07	<1.3	-
Anti-CLβ2GPI	<1.3	<3.5	IU/mL

A head CT scan showed no abnormalities that may explain impaired consciousness. The patient was diagnosed with iTTP based on a PLASMIC score [13] of 6 points, indicating a 66%-82% probability of significantly reduced ADAMTS13 activity. The results of ADAMTS13 activity and inhibitor, which were submitted at the time of admission, became available on the seventh day of hospitalization. Plasma ADAMTS13 activity, measured by enzyme-linked immunosorbent assay, was found to be reduced to 8%, and ADAMTS13 inhibitor was detected at a level of 1.9 Bethesda units (BU)/mL, confirming the diagnosis.

**Figure 2:**
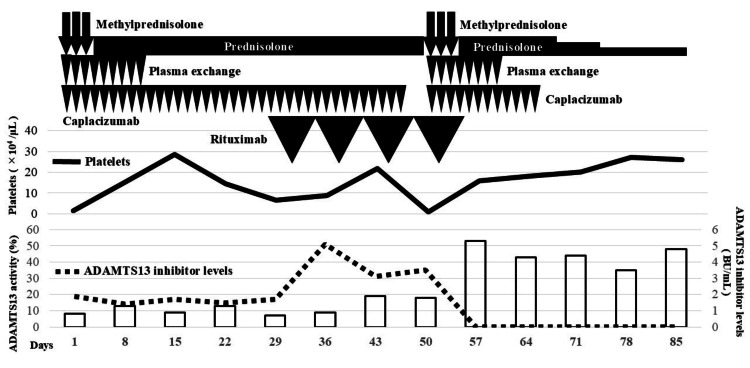
Clinical course The patient was initially treated with mPSL pulse therapy followed by daily prednisolone. PE and caplacizumab were simultaneously initiated. Due to a refractory course, rituximab was added on day 31. Relapse occurred on day 51, prompting the reinitiation of mPSL pulse therapy, PE, and caplacizumab. On day 57, the disappearance of the ADAMTS13 inhibitor and the recovery of ADAMTS13 activity were confirmed mPSL: methylprednisolone; ADAMTS13: antithrombospondin type 1 motif 13; PE: plasma exchange

Figure 2 shows the treatment course, including temporal changes in the platelet count, ADAMTS13 inhibitor levels, and ADAMTS13 activity. The patient initially received daily PE, caplacizumab, and pulsed corticosteroid therapy (methylprednisolone, mPSL, 1,000 mg/day intravenously for three days), followed by oral prednisolone therapy (0.5 mg/kg/day). PE was performed via a blood access catheter using fresh frozen plasma equal to the patient’s circulating plasma volume as the replacement solution. Throughout the clinical course, 2,800 mL of red blood cells was transfused; however, no platelet concentrates were administered. PE was continued daily until two days after the platelet count exceeded 150,000/μL, totaling nine sessions. Caplacizumab and oral prednisolone (0.5 mg/kg/day) were continued thereafter. Although the clinical signs of TTP, such as fever, impaired consciousness, hemolytic anemia, and thrombocytopenia, improved after the initiation of treatment, the ADAMTS13 inhibitor persisted, and ADAMTS13 activity remained suppressed. Therefore, it was impossible to discontinue caplacizumab, and the oral prednisolone dose remained unchanged.

On day 31, the case was deemed refractory due to the persistent presence of the ADAMTS13 inhibitor and the lack of recovery in ADAMTS13 activity, and rituximab (375 mg/m², administered weekly for four weeks) was initiated. Although the ADAMTS13 inhibitor persisted after initiating rituximab, ADAMTS13 activity gradually increased. On day 47, caplacizumab was temporarily discontinued at the patient's request, as the patient, despite being asymptomatic, expressed concerns about the continuous use of this high-cost medication. On day 51, the platelet count significantly decreased to 12,000/μL. No clinical signs of TTP, aside from thrombocytopenia, were observed. The thrombocytopenia decreased shortly after the discontinuation of caplacizumab, combined with an ADAMTS13 inhibitor titer of 3.5 BU/mL and ADAMTS13 activity of 18%, which indicated insufficient recovery; diagnosis of relapse was made. Relapse treatment included restarting caplacizumab and resuming daily PE at the patient’s circulating plasma volume. Pulse mPSL (1,000 mg/day intravenously) was given for three days, followed by oral prednisolone (0.5 mg/kg/day). The only difference from the initial treatment was the continuation of rituximab therapy, including administering the fourth dose. During relapse, PE was performed eight times and discontinued once the platelet count stabilized. The disappearance of the ADAMTS13 inhibitor and the recovery of ADAMTS13 activity were subsequently confirmed, and the patient achieved remission on day 57. Caplacizumab was discontinued, and oral prednisolone was tapered after remission. After achieving remission, the prednisolone dose was tapered based on ADAMTS13 activity and inhibitor levels, starting from 0.5 mg/kg/day and decreasing by 5 mg per week while ensuring the absence of TTP recurrence. During hospitalization, a maintenance dose of 10 mg/day was administered. TTP did not recur during the follow-up, and the patient was discharged on day 100. Outpatient follow-up continued after discharge, with no relapse being reported seven months into remission as of December 2024.

## Discussion

This case report describes a patient with refractory TTP following SARS-CoV-2 infection who was successfully treated with caplacizumab, rituximab, PE, and steroids. Second-line treatment with rituximab for relapsed refractory TTP typically requires approximately 14 days to show efficacy [2,9,10], though responses may vary. Continued PE, corticosteroids, and caplacizumab are essential for patient stability during this period. Caplacizumab accelerates platelet recovery and reduces PE sessions but may delay ADAMTS13 activity recovery, potentially postponing optimal rituximab administration.

Rituximab, a monoclonal antibody targeting CD20, depletes B lymphocytes and suppresses ADAMTS13 inhibitor production [14,15]. It is widely used in Japan as a second-line treatment for refractory TTP [2]. PE may induce "ADAMTS13 inhibitor boosting," where inhibitor levels rise upon ADAMTS13 administration [16]. PE alone may not suppress inhibitor activity, necessitating rituximab combination therapy [17]. Rituximab effectively reduces inhibitors, but therapeutic effects manifest after 10-14 days, requiring concurrent PE, corticosteroids, and caplacizumab to stabilize the acute phase and prevent complications. This combination ensures effective ADAMTS13 inhibitor suppression while maintaining adequate ADAMTS13 activity.

In this case, rituximab was administered as second-line treatment, but ADAMTS13 activity recovery and inhibitor disappearance occurred 34 days after initiation, longer than the typical 10-14 days [2,9,10]. This delay highlights the variability in treatment response. Rituximab depletes B cells via complement-dependent and antibody-dependent cytotoxicity, reducing inhibitor production [14,15]. However, its effects are not immediate, particularly in highly refractory cases. A delay in rituximab initiation following first-line therapy failure may have contributed to the prolonged response. In Japan, rituximab use in the acute phase of TTP is restricted by the national health insurance system, limiting its early administration.

The standard treatment for TTP includes PE and corticosteroids [3,18]. The 2020 International Society on Thrombosis and Hemostasis guidelines recommend adding caplacizumab, a nanobody that inhibits thrombus formation by targeting the interaction between platelets and VWF [6,8]. When used with PE and corticosteroids, it significantly shortens the time to platelet normalization, facilitates early symptom resolution, and reduces PE duration. Additionally, caplacizumab is the first treatment other than PE shown to reduce TTP mortality [7,8]. In this case, caplacizumab, in the initial treatment, temporarily restored platelet counts and allowed PE discontinuation, suggesting its effectiveness even in refractory cases.

However, caplacizumab use may delay ADAMTS13 activity recovery due to reduced PE duration [12,19]. PE replenishes ADAMTS13 and removes inhibitors and ultralarge von Willebrand factor multimers [18]. Shortened PE duration limits these effects, potentially prolonging ADAMTS13 activity recovery [12]. In this case, an insufficient PE duration was likely a factor in delayed ADAMTS13 activity recovery. Additionally, a rapid platelet decline and TTP recurrence followed temporary caplacizumab discontinuation, undertaken due to prolonged treatment duration. This case underscores the difficulty of discontinuing caplacizumab when ADAMTS13 inhibitors persist, and ADAMTS13 activity remains insufficient.

Another potential cause of delayed ADAMTS13 activity recovery may be the timing of rituximab initiation [12]. Initial therapy relies on PE and corticosteroids to suppress ADAMTS13 inhibitors [18]. In refractory cases, intensified immunosuppressive therapy with rituximab is required [9,10]. However, while caplacizumab facilitates early platelet recovery and PE duration reduction, it may inadvertently delay rituximab initiation, which is essential for eliminating ADAMTS13 inhibitors [12]. In this case, ADAMTS13 inhibitors persisted despite corticosteroid continuation after PE cessation. The disappearance of inhibitors was observed only after rituximab administration, suggesting that ADAMTS13 recovery was directly attributable to rituximab’s therapeutic effects.

This case highlights the complexities of managing refractory TTP, particularly the interactions between PE, caplacizumab, and rituximab. While caplacizumab offers rapid platelet recovery and reduces PE burden, it may also limit ADAMTS13 replenishment, affecting treatment response. Rituximab remains essential for sustained ADAMTS13 activity restoration, but its delayed initiation can prolong disease resolution. Optimizing treatment timing, particularly balancing caplacizumab discontinuation and rituximab administration, is crucial to improving outcomes in refractory TTP cases.

## Conclusions

While rituximab is used as a second-line treatment for refractory TTP, it may take several weeks, longer than previously reported, for its therapeutic effects to manifest. This is relevant when caplacizumab is used concurrently because the shortened duration of PE or delayed initiation of rituximab may delay the recovery of ADAMTS13 activity. It is important to note that no definitive third-line treatment has been established for refractory TTP. Therefore, in cases of relapsed or refractory TTP treated with rituximab and caplacizumab, clinicians need to exercise caution. Even if ADAMTS13 inhibitors persist and the recovery of ADAMTS13 activity is delayed following the cessation of PE, a premature attempt at third-line therapy needs to be avoided. Careful observations over several weeks are recommended to allow a sufficient time for the therapeutic effects of rituximab to manifest.
